# Role of hole confinement in the recombination properties of InGaN quantum structures

**DOI:** 10.1038/s41598-019-45218-8

**Published:** 2019-06-21

**Authors:** M. Anikeeva, M. Albrecht, F. Mahler, J. W. Tomm, L. Lymperakis, C. Chèze, R. Calarco, J. Neugebauer, T. Schulz

**Affiliations:** 1 Leibniz-Institut für Kristallzüchtung, Berlin, Germany; 2 Max Born Institute for Nonlinear Optics and Short Pulse Spectroscopy, Berlin, Germany; 3 Max-Planck-Institut für Eisenforschung GmbH, Düsseldorf, Germany; 4 Paul-Drude-Institute of Solid-State Electronics, Berlin, Germany

**Keywords:** Materials for optics, Nanoscale materials, Optical materials and structures

## Abstract

We study the isolated contribution of hole localization for well-known charge carrier recombination properties observed in conventional, polar InGaN quantum wells (QWs). This involves the interplay of charge carrier localization and non-radiative transitions, a non-exponential decay of the emission and a specific temperature dependence of the emission, denoted as “s-shape”. We investigate two dimensional In_0.25_Ga_0.75_N QWs of single monolayer (ML) thickness, stacked in a superlattice with GaN barriers of 6, 12, 25 and 50 MLs. Our results are based on scanning and high-resolution transmission electron microscopy (STEM and HR-TEM), continuous-wave (CW) and time-resolved photoluminescence (TRPL) measurements as well as density functional theory (DFT) calculations. We show that the recombination processes in our structures are not affected by polarization fields and electron localization. Nevertheless, we observe all the aforementioned recombination properties typically found in standard polar InGaN quantum wells. Via decreasing the GaN barrier width to 6 MLs and below, the localization of holes in our QWs is strongly reduced. This enhances the influence of non-radiative recombination, resulting in a decreased lifetime of the emission, a weaker spectral dependence of the decay time and a reduced *s-*shape of the emission peak. These findings suggest that single exponential decay observed in non-polar QWs might be related to an increasing influence of non-radiative transitions.

## Introduction

The growth of high quality InGaN quantum wells has been one of the key inventions that paved the way to efficient group III-Nitride based light emitting diodes^1–3^. Carrier localization crucially controls the properties and efficiencies in optoelectronic devices^4,5^. It has early been hypothesized that carrier confinement in the InGaN alloy could explain the high radiative efficiency of quantum structures despite the high densities of threading dislocations^6^. Bellaiche *et al*.^7^ were the first to emphasize that localization of holes might be an intrinsic property even of a statistical InGaN alloy.

A large variety of mechanisms have been proposed to govern charge carrier localization such as alloy fluctuations, QW thickness variations and polarization fields^4,8–11^. A non-exponential decay of the luminescence with increasing decay times towards the lower energy side of the emission spectrum, as well as a temperature dependence of the emission, typically denoted as *s*-shape, were seen as evidence for carrier localization in polar InGaN^12–15^. Several models have been proposed to explain the observed phenomena based on the: (i) spatial separation of localized, both, electrons and holes^13^; (ii) variation of the local polarization fields due to the compositional fluctuations^14,15^; (iii) charge carrier transfer to lower energy states^16^. All these diverse models were proposed for bulk or few nm thick QW systems, where various localization phenomena interact in a complex and dynamic way. Therefore, in this paper we study localization phenomena in polar thin InGaN films realized in the form of a digital alloy. This reduces the complexity of the underlying system allowing to decouple several effects governing charge carrier localization.

Our system consists of single ML thick InGaN quantum wells separated by GaN barriers ranging from 50 MLs to 6 MLs, so we can control the charge carrier confinement as done for group III-Arsenide semiconductors^17–20^. The growth of such QWs is self-limited to one monolayer with a mean In content of around 25%^21,22^. Due to the small width of the quantum well, the influence of the quantum confined Stark effect (QCSE) on the emission is negligible^11^. By combining quantitative transmission electron microscopy (TEM), cw-photoluminescence (cw-PL), and time-resolved PL (TRPL) experiments with density functional theory (DFT) calculations, we get insights into the charge carrier confinement and the recombination mechanism.

We show that single ML thick In_0.25_Ga_0.75_N QWs with thick GaN barriers exhibit high degree of localization only for hole states for GaN barrier widths above 12 MLs. In contrast, electrons are mostly delocalized over the barrier region for all thicknesses. Our main findings are:

(i) Several optical properties, i.e. a non-exponential decay and an *s*-shape temperature dependence of the emission peak positions, are very similar to conventional several nm thick polar InGaN quantum wells. Thus, we exclude that these phenomena are necessarily connected to the QCSE, electron localization, QW width- or gross In fluctuations such as In rich clusters.

(ii) Non-radiative recombination can be enhanced by decreasing the hole localization via reducing the GaN barrier width. As a consequence, we obtain a more single exponential decay and a less pronounced spectral dependence of the decay time, as well as a weaker *s*-shape of the emission with temperature. We propose that the single-exponential decay in non-polar QWs is, thus, due to the generally much faster recombination in such systems.

## Results

### Structural characterization

**Figure 1:**
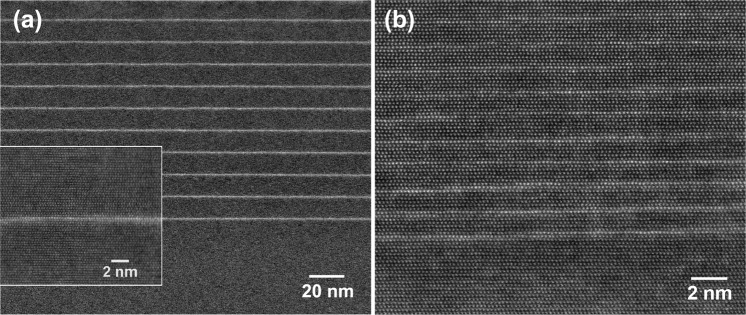
STEM-HAADF images of the (**a**) 50 ML and (**b**) 6 MLs barrier samples. The inset shows a higher magnification of 1 ML (In,Ga)N QW for the 50 MLs sample. An appearing growth step on the left part of the HR-TEM image is visible.

A series of four SL samples each consisting of ten periods of polar InGaN QWs separated by GaN barriers of different thickness were grown by molecular beam epitaxy (MBE) at 550 °C^23^. Composition and thickness of the InGaN layers and the GaN barriers are quantified by TEM throughout the entire sample series. High resolution STEM-HAADF investigations reveal GaN barrier thicknesses of 50 MLs, 25 MLs, 12 MLs and 6 MLs with an accuracy of ±1 ML. As an example, Fig. 1(a,b) display STEM–HAADF images in the <11–20> zone axis of samples with the thickest (50 MLs) and the thinnest GaN barrier (6 MLs), respectively. A typical high-resolution STEM image of an InGaN QW of the structure with 50 MLs GaN barrier is shown in an inset of Fig. 1(a) at a similar magnification as compared to Fig. 1(b).

The InGaN MLs appear at a higher HAADF intensity compared to the surrounding GaN due to their higher mean atomic number. All InGaN layers are coherent to the surrounding GaN matrix and reveal abrupt interfaces to the GaN barriers. Note the apparent increase of the QW thickness in the STEM images taken under high magnification is due to surface steps partially lying inclined to the electron beam. To exclude such effects, we have performed HR-TEM analysis for very thin specimen thicknesses, i.e ~10 nm, confirming a QW thickness of a single ML throughout the entire series Fig. 1(a). This experimental observation is supported by the theoretical findings revealing that a self-limitation mechanism prevents the formation of a second monolayer under the utilized growth conditions, as published in ref.^21^.

**Figure 2:**
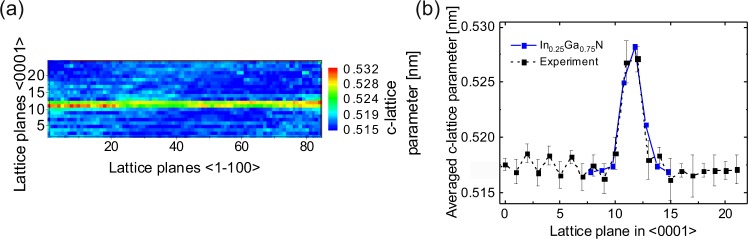
(**a**) Color coded c-lattice parameter map showing a single InGaN ML. (**b**) Laterally averaged c-lattice parameters and standard deviations for the measured sample and the calculated supercell (blue solid line) consisting of an InGaN ML with a mean In content of 25%.

For quantifying the indium content, we measured the c-lattice parameter in a series of 30 HR-TEM images as described in ref.^24^. The structure was stable against the beam damage as monitored during the HR-TEM session. The color-coded c-lattice parameter map of the InGaN ML region for the sample with 50 MLs GaN barrier is displayed in Fig. 2(a). A single InGaN ML induces an increase of 2–3 neighboring c-lattice parameters along the <0001> direction as a consequence of measuring a full c-lattice spacing. A slight variation of the measured c-lattice parameters within the ML is observed, which indicates a laterally inhomogeneous distribution of In. To quantify the mean indium content of the QW, we averaged the measured c-lattice parameters laterally, as displayed in Fig. 2(b) (dashed line).

A comparison of the experimentally measured profile of the laterally averaged c-lattice parameters (dashed curve), shows very good agreement with a simulated profile of an InGaN ML with an In content of 25% (solid curve) as displayed in Fig. 2(b). Very similar compositions were obtained for all QWs throughout the entire sample series. From the standard deviation of the experimentally measured lattice parameter fluctuations in GaN, we deduce a measuring precision with a standard deviation of ~1.3 pm. Assuming a linear interpolation of the c-lattice parameter between GaN and InGaN ML with 25% of In, this approximately yields a composition quantification precision of ±3%.

### Optical properties

**Figure 3:**
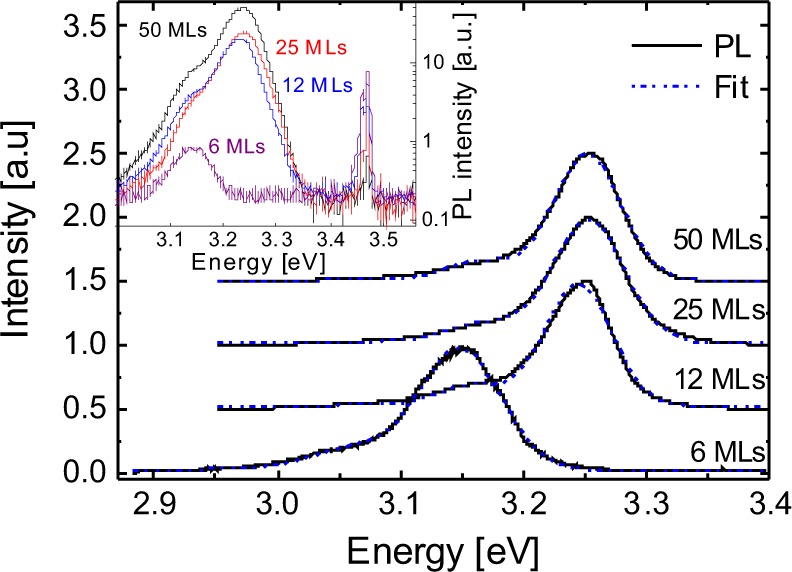
Normalized photoluminescence spectra of all SL samples measured at 10 K. Gaussian fitting of the spectra is indicated by blue dashed curve. The original experimental data are shown on the inset. The fitting parameters are summarized in Table 1.

Low temperature (i.e. 10 K) cw-PL and time-resolved PL studies were carried out for all samples, as displayed in Fig. 3. All spectra were normalized to their respective SL emission peaks located between 3.1 eV and 3.3 eV. The inset in Fig. 3 shows the respective full spectra including the emission from the underlying GaN template in a semi-logarithmic scale. The emission bands from the InGaN QWs are accompanied by a smaller shoulder appearing about 90 meV towards the lower energy side of the SL main peak that can be attributed to the first longitudinal optical phonon replica^25^. Each spectrum was fitted by two Gaussian functions, with the constraints of (i) similar full width at half maxima (FWHM) and (ii) a fixed peak position of the lower energy emission band at 90 meV below the main emission peak. To compare the SL emission yields quantitatively, we have normalized the integrated emission intensity by the proportion of light, which is absorbed within each SL stack, respectively. The latter was calculated employing the Beer-Lambert law using a mean absorption coefficient of α = 1.0 × 10^5^ cm^−1^ corresponding to an excitation energy of 3.8 eV, for both, the InGaN and GaN layers^26^. All resulting parameters are summarized in Table 1.

**Table 1:** Parameters of the PL spectra derived from Gaussian fitting.

Barrier width [MLs]	Peak energy [eV]	FWHM [meV]	Integrated intensity (absorption corrected) [a.u.]
50	3.25	66	5.6
25	3.25	70	4.0
12	3.24	65	5.8
6	3.15	77	0.3

The emission bands of the two samples with GaN barriers of 50 and 25 MLs are practically identical with respect to their peak emission energies and FWHM. FWHM of all samples are in the range of 65–77 meV. However, for smaller barrier widths, the emission energy shifts towards lower values, whereby the most pronounced redshift is observed for the sample with 6 MLs GaN barrier. This is accompanied by a reduced luminescence yield, which is about 17 times lower as compared to the samples with thicker barriers.

To account for possible changes of polarization ratio due to the modification of the band structure for the very thin barrier SL, we have additionally performed PL experiments using excition along the c-direction and detection in a 90° orientation along the a-direction similar to ref.^27^. No substantial differences in the results between these two experiments were revealed, i.e. the thinnest barrier sample showed a very low luminescence efficiency. The much lower luminescence yield of the 6 MLs barrier sample was additionally confirmed by means of quasi-resonant PL measurements, using an excitation wavelength below the bandgap of GaN^28^. Moreover, this effect does not depend on the thickness of a GaN cap layer as confirmed by PL measurements of an additional sample with 6 MLs GaN barrier capped with 40 nm of GaN (not shown here).

The pronounced differences of the emission peak and luminescence yields for SLs with very thin (6 MLs) barriers suggest a change of the SL bandgap, as well as the underlying recombination dynamics. To exclude a contribution of the QCSE in this context, we have performed power dependent PL experiments using excitation powers from 50 µW to 5500 µW revealing a slight redshift of the peak emission (see Supplemental Material Fig. S1). This effect is associated with the well-known band gap renormalization effect due to the high amounts of free charge carriers^29,30^. A measurable influence of the QCSE, which should result in a blueshift with increasing excitation power see e.g. refs^31,32^ is, thus, not found.

### Recombination dynamics

**Figure 4:**
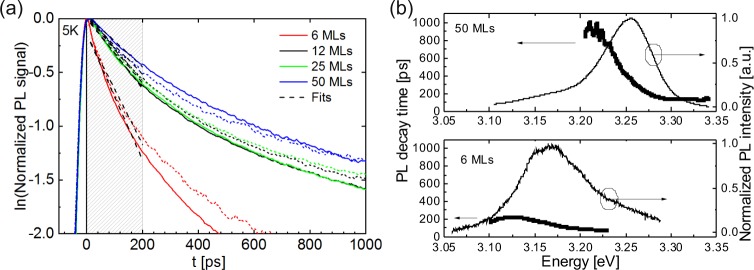
(**a**) Transients taken for the 50, 25, 12 and 6 MLs structures measured under two different excitation powers at 5 K. Dotted/solid curves are spectra taken under lower/higher powers, respectively. Light grey dashed lines are the fits applied in the marked area. (**b**) Spectral dependences of the initial decay time of two samples with 6 MLs and 50 MLs barriers, respectively along with their normalized PL emission.

To study the influence of the GaN barrier thickness on the recombination dynamics we have carried out TRPL experiments. Figure 4(a) displays the transients for all four samples using a 4.79 eV laser excitation energy at a temperature of 5 K. All transients were extracted using a spectral window of about ±40 meV around the respective emission peak position. To exclude effects related to different population densities, excitation powers were adapted for each sample with respect to the total absorption in the structures; therefore, the proportion of absorbed energy remains constant for the different SL stack widths. Using α (4.79 eV) = 2.0 × 10^5^ cm^−1^ yields 9.8 mW (6 MLs), 6.16 mW (12 MLs), 4.17 mW (25 MLs) and 3.23 mW (50 MLs). Moreover, to determine the contribution of the GaN barriers to the non-equilibrium carrier population of the QWs, TRPL measurements for each sample were carried out in a high and low excitation power regime, varied by one order of magnitude. These issues are discussed in detail in the Supplemental Material.

As can be seen in Fig. 4(a) all specimens exhibit a non-exponential decay behavior. The decay time was determined from a linear fit in the semi-logarithmic plot in a time window ranging from 0 to 200 ps depicted by the gray shaded region in Fig. 4(a), denoted as the ‘initial decay time’ in the following discussion. The results are summarized in Table 2. Samples with 50 MLs, 25 MLs and 12 MLs barriers exhibit similar decay behavior, whereas the structure with the thinnest barrier (6 MLs) shows a much faster decay for both excitation powers. Since all samples are, aside from the barrier width, structurally identical, this points to a change of the charge carrier localization as a consequence of the smaller barrier width.

**Table 2:** Initial decay times of SLs with different barriers obtained from the fits of the curves measured under different powers.

Carrier density per QW, cm^−2^	6 MLs [ps]	12 MLs [ps]	25 MLs [ps]	50 MLs [ps]
5.5 × 10^8^	195 ± 10	326 ± 6	324 ± 8	377 ± 12
5.5 × 10^9^	171 ± 7	303 ± 7	314 ± 10	422 ± 9

Exact values of excitation powers for each SL are mentioned above in the text. The double standard deviation obtained from the fits are shown as the errors of the experimental values. Here we assume that all carriers generated in the barriers and quantum wells contribute to the carrier density in the quantum wells (for more details, see Supplemental Material).

Figure 4(b) (top and bottom) displays the spectral dependence of the initial PL decay time together with the time integrated PL spectrum for the 50 MLs and the 6 MLs GaN barrier, respectively. Although, in both cases we observe an increase of the initial decay time for decreasing emission energies, for the 6 MLs GaN barrier case this effect is much less pronounced.

**Figure 5:**
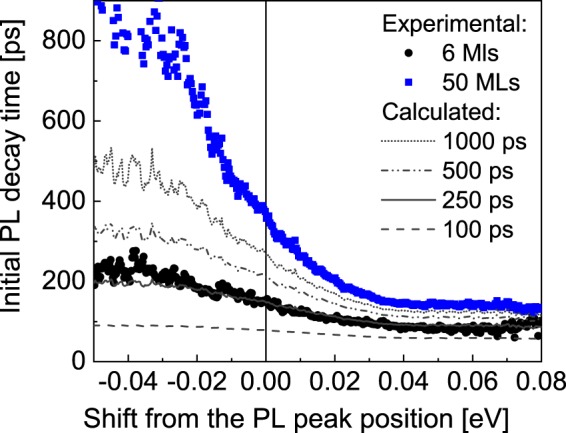
Decay time spectra (black dots) of two samples with 50 MLs and 6 MLs GaN barriers centered to their peak emission positions (chosen as 0 eV). Grey curves represent the calculated spectral dependences for different non-radiative recombination times.

In the following we examine whether the faster recombination and the less pronounced spectral dependence of the initial decay time for the 6 MLs GaN barrier sample can be explained by an increased contribution of non-radiative transitions. In Fig. 5 we plot the initial decay times of the 50 MLs and the 6 MLs GaN barrier sample aligned to their respective cw-PL emission peak position.

Assuming an energy dependent radiative recombination time *τ*_*rad*_(*E*), the total recombination rate *τ* is given by

1 $$\frac{1}{\tau (E)}=\frac{1}{{\tau }_{rad}(E)}+\frac{1}{{\tau }_{nrad}},$$

with *τ*_*nrad*_ being the non-radiative decay time.

A faster luminescence decay of the 6 MLs SL recombination can be either due to a reduced *τ*_*rad*_(*E*) or *τ*_*nrad*_. Keeping in mind that our PL experiments revealed a substantial decrease of the total PL yield for the 6 MLs barrier case, this suggests a decrease of *τ*_*nrad*_. Using the measured initial decay times of the 50 MLs barrier sample as input values for the *τ*_*rad*_(*E*), we have gradually enhanced non-radiative contributions by introducing an energy-independent *τ*_*nrad*_ with decreasing values. The curves calculated using Eq. (1) for different non-radiative decay times of *τ*_*nrad*_ between 1000 ps to 100 ps, are also plotted in Fig. 5. An almost perfect agreement between the spectral dependences of the 50 MLs and the 6 MLs barrier sample is obtained by adding for the latter sample a non-radiative recombination channel with *τ*_*nrad*_ = 25  ps. Thus, we conclude that the underlying energy dependent radiative recombination process is identical for all samples, while only the impact of non-radiative transitions is strongly enhanced for the 6 MLs GaN barrier sample.

### DFT calculations

To understand the origin of the increased contribution of non-radiative transitions for the 6 MLs GaN barrier SL we have investigated the electronic structure, effective masses and the carrier wavefunctions of InGaN/MLs GaN SLs by means of DFT calculations. The supercells contain a single In_0.25_Ga_0.75_N ML with GaN barriers ranging from 1 to 19 MLs and have a lateral extension of 2 × 2 nm ?. The calculated bandgaps of the SLs show good agreement with the measured emission peak energies (see Supplemental material Fig. S4).

**Figure 6:**
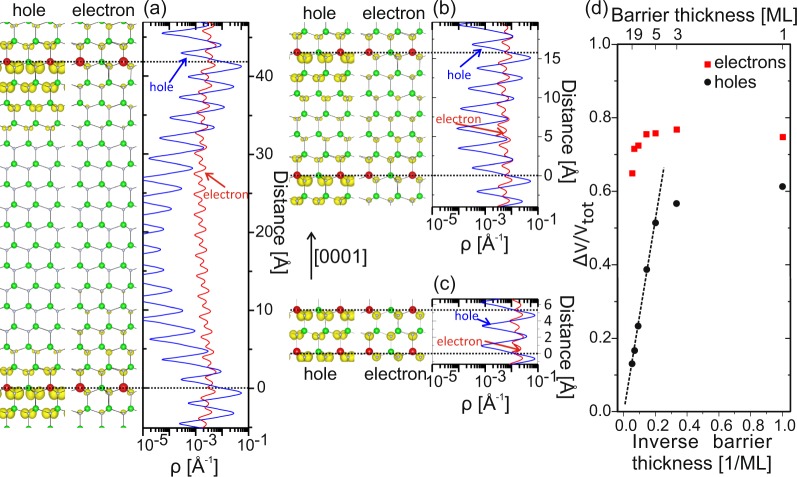
Partial charge densities of the VBM (holes) and CBM (electrons) (left) and the corresponding planar averaged profiles (right) for (**a**) 1:15, (**b**) 1:5 and (**c**) 1:1 In0.25Ga0.75N/GaN SLs. Large red and green circles indicate In and Ga atoms, respectively. Small gray balls indicate the N atoms. (**d**) Ratio of the SL volume that localizes 80% of the VBM (black circles) and CBM (red squares) charge densities as function of the barrier thickness. The dashed line in (**d**) is a linear fit to the data with barrier thicknesses larger than 5 MLs.

The charge density at the valence band maximum (VBM) and conduction band minimum (CBM) for the SLs with barrier thicknesses of 15 MLs, 5 MLs and 1 ML, are displayed in Fig. 6(a–c), respectively. For the 15:1 SL the *p*-state of the VBM is mainly localized within 3 MLs around the QW. Reducing the GaN barrier to 5:1 SL, increases the hole charge density in GaN barriers, which becomes most pronounced for the 1:1 SL. Turning to the electron wavefunction, one finds that the charge density of the *s*-states at the CBM are practically uniformly distributed and, therefore, delocalized along the <0001> direction for all calculated SLs. For instance, for the SL with the 15 MLs barrier the electron charge density is only by a factor of 2 smaller in the middle of the barrier than in the QW. Compared to that the hole charge density changes in the same regions by 3 orders of magnitude lower (see Fig. 6(a), right).

To provide a more quantitative description of the charge carrier localization, we have calculated the ratio Δ*V*_*i*_/*V*_*i*_ for different SLs, where Δ*V*_*i*_ denotes the volume of the SL supercell that localizes 80% of the charge carrier density and *V*_*i*_ corresponds to the total supercell volume. Here, smaller ratios indicate stronger localization of the charge carriers inside a limited volume, whereas the ${\rm{\Delta }}{V}_{i}/{V}_{i}\to 1$ implies that the charge carrier wavefunctions are distributed uniformly within the entire supercell. As can be seen in Fig. 6(d) the localization of holes and electrons show a very different behavior. For the holes, Δ*V*_*i*_/*V*_*i*_ is inversely proportional for SL barriers above 5 MLs, which implies that the volume that localizes 80% of the holes does not change for barrier widths above 5 MLs. This indicates a strong hole localization in the vicinity of the QW for such SLs. From the slope of the dashed line in the Fig. 6(d) we calculate the thickness of the slab around the InGaN QW that confines 80% of the hole charge equals ≈2.6 MLs, i.e. ≈13.8 Å. For SLs with barriers thinner than 5 MLs the ratios deviate from the linear dependence and reach a value of ≈0.6 for the 1:1 SL. This corresponds to a strongly reduced localization of the hole charge implying strong overlap of the hole wavefunctions from neighboring QWs. In contrast to the holes, the localization of the electronic charge densities shows a weak confinement for all calculated thicknesses. For thin barriers less than 5 MLs ≈75% of the SL volume is required to localize 80% of the electrons which only slightly reduces to ≈72% for the 15 MLs GaN barrier SL.

We note that the aforementioned calculations do not include the effect of the attractive electron hole Coulomb interactions. We have investigated their influence on the carrier confinement by explicitly introducing a hole in the QWs of the 1:7 and 1:11 SLs. Our calculations show that the attractive Coulomb interactions have no qualitative and only a minor quantitative effect on carrier localization. More specifically, we find that the ratio of the SL volume that localizes 80% of the electron charge density is 76% and 73% without Coulomb interactions and 74% and 68% with Coulomb interactions for the 1:7 and 1:11 SLs, respectively.

The different electron and hole localization properties strongly influence the corresponding effective masses and hence the charge carriers mobility. According to our calculations, the effective masses of holes are strongly dependent on the barrier thickness, i.e. they monotonously increase with the barrier width (see Supplemental Material Fig. S5). In contrast, the electron effective masses are insensitive to the barrier width. Our calculations allows a distinction of the considered structures into thick barrier SLs where holes are strongly localized in the vicinity of the QW, and thin barrier SLs where holes are becoming delocalized via interwell coupling of the wavefunctions. This practically denotes the transition from a localized hole - to a continuum state, which makes the SL resemble a quasi-ordered InGaN alloy along the <0001> direction. Electrons are generally delocalized for all considered structures which is independent on the barrier width.

## Discussion

We investigated the structural and optical properties of the SLs with varying GaN barrier widths. Structural investigations consistently reveal single ML thick In_0.25_Ga_0.75_N QWs with GaN barriers of 50 MLs, 25 MLs, 12 MLs and 6 MLs. Optical measurements confirm the absence of the QCSE for all discussed SLs (see Supplemental Material Fig. S1) which is expected for thin QWs^11^. This makes the localization of charge carriers solely dependent on the thickness of the GaN barriers.

Emission intensities, peak emission energies (see Table 1) and recombination dynamics (see Fig. 4) are very similar for all *thick* barrier SLs of 50 MLs, 25 MLs and 12 MLs, while noticable changes only occurr for thin GaN barriers of 6 MLs. In the following, we will discuss the optical properties in the context of our DFT calculations. We note, that the DFT data only allow to assess the charge carrier localization along the <0001> directions (out-of plane), while localization and spatial extents of wavefunctions along the in-plane directions of the SLs cannot be evaluated due to the geometry of our supercells. According to our calculations, *thick* barrier SLs (50 MLs, 25 MLs, 12 MLs) allow an efficient localization of holes within a small range of 1.3 nm around the QW, while electrons are practically delocalized along the entire QW stack. Accordingly, we expect similar recombination properties for all such samples. In fact, the emission of all *thick* barrier SLs occurs around 3.25 eV with comparable luminescence yields. Such emission energies are within the predicted range for single ML-thick QWs with In contents of around 25%^33^ and agree well with other samples grown under comparable conditions^34^. Also the recombination dynamics, revealing a non-exponential decay with increasing decay times towards lower energies are very similar for all thick barrier SLs.

In contrast, decreasing the barrier thickness to 6 MLs leads, according to our DFT data, to a coupling of the hole wavefunction which reduces the hole localization in the vicinity of the QW. As a consequence, this should affect prominent recombination properties. Indeed, one observes a red shift of the emission, which follows the calculated bandgap energy (see Supplemental Material Fig. S4). Moreover, for this sample we find a decreasing decay time which is accompanied by a decrease of the luminescence yield. We relate these effects to an increased contribution of non-radiative transitions, as a result of the decreased hole localization in the vicinity of the QWs. This can be shown by adding an energy independent non-radiative recombination channel to the spectral dependence of the decay time measured for the 50 MLs GaN barrier SL, as shown in Fig. 5, which provides excellent agreement with the 6 MLs barrier sample. Hence, the underlying energy dependent radiative recombination process, described by *τ*_*rad*_(*E*) in Eq. (1), does not change. Indeed, we have observed experimentally a strong decrease of the emission intensity for the 6 MLs thick barrier sample in PL measurements for different excitation and detection conditions. Thus, reducing the hole localization enhances their probability of reaching non-radiative recombination centers in the GaN barriers^23^. Since the GaN barriers were grown at 550 °C, optimized for achieving high In contents in the QWs, it is far below the optimum for GaN and, thus, results in a high number of point defects. An investigation of the exact nature of these point defects is beyond the scope of this paper.

The increasing overlap integral of the electron and hole wavefunctions for decreasing GaN barriers which, according to other theoretical studies, should promote radiative recombination, does not seem to overcompensate this effect^35^. We note that a similar decrease of the luminescence yield for thin barrier SLs was observed in *nominal* InN/GaN superlattices^36^, which possibly indicates its relation to the same phenomenon. Thus, we have shown for the first time a clear experimental evidence of hole coupling in QWs, which confirms their strong spatial localization in the QWs to below 2 nm. Reducing the hole localization in a SL with GaN barriers <12 MLs promotes non-radiative transitions while the underlying general radiative recombination mechanism remains unaffected.

In the last part, we will discuss some general implications on the recombination dynamics in conventional, several nm thick QWs. First, with respect to the recombination dynamics, we notice distinct similarities to several nm thick polar InGaN QWs, i.e. a non-exponential decay with increasing decay times towards lower energies and an *s*-shape temperature dependence of the peak position.

A non-exponential decay has been explained by means of an in-plane spatial separation of individually localized electrons and holes in the context of a two dimensional donor-acceptor pair recombination^13^. A similar model has been discussed in the work of Feix *et al*.^28^ observing a power law decay for a similar set of samples measured on a long range time scale (up to 30 ns). However, strong in-plane localization of electrons within the In_0.25_Ga_0.75_N QW is unlikely for our system since the InGaN composition variations are barely above the measuring precision of ±3% of In, suggesting a random alloy. In addition, the self-limiting growth process^21,22^ impedes any QW width fluctuations which could promote additional in-plane localization of electrons^37^. We note that compared to several nm thick non-polar InGaN QWs where a single exponential decay is observed^38^ the compositional fluctuations seen by electrons in our system are negligibly small. We, thus conclude that an in-plane spatial separation of electron and hole pairs for explaining the recombination properties seems not applicable to our system.

Another model explains a non-exponential decay, with increasing decay times towards lower energies to originate from the out of-plane spatial separation of charge carriers due to a local QCSE^14^. However, since we practically observe the same phenomena in our system where the QCSE is irrelevant for the recombination, which particularly accounts for the high charge carrier densities as present in our experiments, we conclude that this model does also not allow to describe the recombination in our system. Note that an increasing decay time towards lower energies is also found in other systems with a negligible influence of the QCSE, i.e. in bulk systems, e.g. InGaAsN^39^ and AlGaN^40^ epilayers.

Alternative models explaining non-exponential decay characteristics involve charge carrier transfer to lower energy states^16,41,42^ or mixed approaches involving both – charge carrier transfer and spatial separation^43^. There, excitons, which preferentially form in our SL system via Coulomb attraction of delocalized electrons and strongly localized holes, can reach lower energy states within the potential landscape via acoustic phonon assisted tunneling. Since such processes may take place at cryogenic temperatures these models are applicable to our system. A careful measurement of the PL rise times, which are expected to increase towards the low energy side of the spectrum due to their delayed population, is required. Indeed, our first comparison of the low and high energy shoulders of the rise times in the TRPL spectra (not shown here) reveals that the latter ones are steeper (i.e. faster). Following these models, the single exponential decay observed in non-polar InGaN QWs^14^ might then be simply related to: (i) the generally faster charge carrier recombination process inhibiting the carrier transfer process and/or (ii) to a less efficient charge carrier transport within a non-polar plane.

For non-polar, randomly distributed several nm thick InGaN alloys the compositional fluctuations are expected to be much larger, where, however, a single exponential decay is observed^38^. Nevertheless, a detailed understanding of the carrier localization would require atomistic calculations of the In_0.25_Ga_0.75_N monolayer including in-plane compositional fluctuations, which is beyond the scope of this paper.

Finally, we will discuss another common characteristic between the described ML-thick QWs and conventional, polar thick QWs, i.e. the temperature dependence of the emission peak in the form of *s*-shape. This phenomenon is characterized by a decreasing emission energy of the peak from around 4 K to 100 K, followed by an increase and again a subsequent decrease up to room temperature^9^. The initial red shift of the emission is often attributed to a thermal redistribution of charge carriers towards lower lying energy states centers within the alloy^44^. Applied to our system, where electron localization is negligible, we, thus, conclude that the initial red shift of the emission, reflects only degree of the hole or exciton localization. Reducing of the hole confinement observed for thin barrier SLs, should decrease the magnitude of this initial shift. Indeed, we observe a larger magnitude of the first red shift between 7 K and 100 K for thick barrier SLs (20 meV) as compared to thin barrier SLs (5 meV) measured by Feix *et al*.^28^. The magnitude of this red shift of the thick barrier SLs is comparable to those observed in conventional polar thick QWs^45^. One has to keep in mind also that the enhanced role of non-radiative recombinations might hamper a direct assessment of the various localization energies of holes (or excitons). The latter factor might explain the absence of an *s*-shape in non-polar InGaN QWs.

## Conclusion

In summary, by investigating the emission properties of single monolayer In_0.25_Ga_0.75_N/GaN SLs in dependence of the barrier thickness, we are able to investigate the impact of hole localization and hole wavefunction coupling. Other factors influencing the emission such as a substantial electron localization, the QCSE, QW width fluctuations or gross In fluctuations are not observed in our system. We find that GaN barriers below 12 MLs trigger hole wavefunction coupling along the <0001> direction, which reduces the out-of plane hole confinement. This, in turn promotes non-radiative transitions which affects the spectral dependence of the initial decay time and the *s*-shape of the temperature dependece of the emission. However, despite the reduced complexity of such SLs we observe very similar optical phenomena as compared to conventional, several nm thick polar QWs: a non-exponential decay of the emission with an increasing decay time towards lower emission energies and *s*-shape temperature dependence of the peak position. Based on these findings we discuss some inconsistencies with existing models in literature based on licalization of electrons and holes. Instead, we propose that a redistribution of holes, or excitons formed by Coulomb attraction of electrons, via e.g. a tunneling process promoted by acoustic phonons causes these effects even at cryogenic temperatures. This would imply that the single exponential decay found in non-polar QWs might then be related to a generally faster recombination process preventing the charge carrier redistribution – or a less efficient redistribution process along a non-polar lattice plane.

## Methods

### TEM investigations and simulations

Structural analyses were carried out using transmission electron microscopy (TEM) and scanning transmission electron microscopy high angle annular dark field (STEM-HAADF) imaging, using an aberration corrected FEI Titan 80–300, operated at 300 kV. For TEM imaging, we used negative spherical aberration imaging conditions as described in ref.^46^. STEM-HAADF measurements were done with an acceptance semi-angle of 35 mrad of the annular dark field detector. The convergence semi-angle was 9 mrad. Cross-sectional samples in <1–100> and <11–20> projections were prepared by mechanical polishing to the thickness approximately 10 µm and then thinned to the 10 nm by the Ar ion milling with a precision ion polishing system (PIPS) from Gatan using acceleration voltages from 4 kV to 0.1 kV.

TEM image simulations were carried out by means of a multislice approach using supercells, relaxed with a modified embedded atom method (MEAM) empirical potential^47,48^. Each supercell had dimensions of 3 nm along the <0001> direction, 1 nm along the <1–100> direction and 3 nm along the <11–20> direction, with the latter being used as the projection direction. All supercells contained a single InGaN monolayer with randomly distributed atoms and different compositions ranging from 17% to 100%. Since the InGaN QW thickness is only 1 ML, thin foil relaxation along the projection direction can be neglected. Thus, periodic boundary conditions are applied along all directions, resulting in a biaxial strain state of the InGaN monolayer. The relaxed supercells were used for TEM image simulations using comparable imaging conditions as in the experiment.

### Optical measurements

Continuous wave (cw)-PL experiments were conducted using the 325 nm line of a HeCd laser. For the detection of the cw-PL signal, we used a 0.75 m Acton spectrometer in combination with a charge coupled device (CCD) camera. TRPL was measured by using a frequency tripled Spectra-Physics Tsunami Ti:sapphire laser at λ = 259 nm (4.79 eV). The repetition rate of the system was 80 MHz with a pulse length ≈ 100 fs and a focus spot diameter of around 100 µm. This yields typical excitation densities of 6*10^11^ photons/cm^2^ per pulse (i.e. 40 W/cm^2^ averaged power) for 259 nm. The PL is analyzed using an Acton SP2300 0.3 m imaging monochromator in combination with a Hamamatsu C5680–21 streak camera with S20 photocathode, operated in synchro-scan mode. The time resolution of the entire system is better than 10 ps (1/e decay). The samples were glued to the cold-head of an optical Helium closed-cycle cryostat, allowing to control the temperature in the range from 5 K to room temperature.

### DFT calculations

Electronic structure calculations have been conducted employing density functional theory (DFT) and the projector augmented-wave (PAW) method^49,50^. The Heyd, Scuseria, and Ernzerhof hybrid functional^51^ was used and the Ga and In 3*d* and 4*d* electrons are treated as core electrons. A plane-wave energy cutoff of 450 eV was used and the Brillouin zone (BZ) was sampled using an equivalent 8 × 8 × 6 Monkhorst-Pack k-point mesh for the unit cell. Single ML In_0.25_Ga_0.75_N QWs were considered biaxially strained to GaN. We modelled the SLs employing 2 × 2 × *m* supercells (m = 2, 4, 6, 8, 12, 16, and 20) consisting of 1 ML In_0.25_Ga_0.75_N embedded in *m-1 MLs of* GaN.

## Supplementary information

Supplemental Material

## Data Availability

The datasets generated and analyzed during the current study are available from the corresponding author on reasonable request.
