# Supplementary material for: Role of hole confinement in the recombination properties of InGaN quantum structures
Source: Sci Rep. 2019 Jun 21;9:9047. doi: 10.1038/s41598-019-45218-8 (PMC6588636; doi:10.1038/s41598-019-45218-8)
Supplement: Supplementary file 1 — Supplemental Material [file 41598_2019_45218_MOESM1_ESM.pdf]

Supplemental material

“Role of hole confinement in recombination properties of InGaN quantum structures.”

M. Anikeeva*[1], M. Albrecht[1], F. Mahler[2], J. W.Tomm[2], L. Lymperakis[3], C. Cheze[4], R. Calarco[4], J. Neugebauer[3] and T. Schulz[1]

[1] *Leibniz-Institut für Kristallzüchtung, Berlin, Germany*
[2] *Max Born Institute for Nonlinear Optics and Short Pulse Spectroscopy, Berlin, Germany*
[3] *Max-Planck- Institute für Eisenforschung, Düsseldorf, Germany*
[4] *Paul-Drude-Institute of Solid-State Electronics, Berlin, Germany*

Power dependence of the QW peak position.

Piezoelectric polarization fields that induce the quantum confined Stark effect (QCSE) are strongly affecting the optical properties in the conventional 2-3 nm thick polar InGaN QW. Previously, it was shown that reducing the width of the well decreases the influence of the QCSE[1]. To validate this assumption for our one monolayer-thick QWs experimentally, we performed the PL measurements under increasing excitation power. We used an excitation energy of 4.79 eV in pulsed mode, which avoids thermal heating effects. The whole series shows a very similar behavior, therefore, we restrict the following discussion to the SLs with 50 and 6 MLs GaN barriers. Excitation densities in the experiment were between 7.5×10^{9} – 4.5×10^{11} photons/cm^{-2} per pulse for the 50 MLs sample and between 3.4×10^{10} - 4.5×10^{11} photons/cm^{-2} per pulse for the 6 MLs structure. This corresponds to a time-averaged excitation power range between 0.15 mW–3 mW. The excitation power dependences of the PL peak positions of the spectra, extracted from a 250 ps time window after the excitation pulse, are presented in Fig. S1.

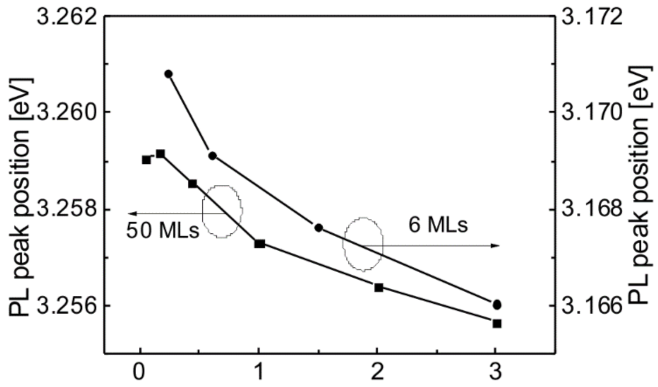

Fig. S1 Peak position extracted from the spectra of the 50 and 6 MLs barrier samples acquired under pulsed excitation with different excitation powers at 5 K.

Both samples reveal a slight redshift of the peak emission for increasing excitation power instead of a blueshift expected for the same power range due to the QCSE and shown in Refs.[5,6]. In our experiments, the total shifts of the emissions are comparable for both samples and are approximately 3.5±1.0 meV and 4.8±1.0 meV for the SLs with 50 and 6 MLs barrier thicknesses, respectively. We associate these redshifts of the PL spectra with the band gap renormalization observed in semiconductors under the presence of high amounts of free charge carriers[2,3]. For the thick polar QWs this effect is hardly observable due to the predominant screening of the piezoelectric polarization under the increased excitation power[4]. Thus, for our structures a measurable influence of the QCSE is not found.

Power dependence of the initial decay.

Additionally, we have performed power dependent TR PL measurements in a wide excitation range from 50 µW to 3000 µW (50 MLs) and 230 – 5500 µW (6 MLs) under 4.79 eV excitation. The decay times in the 0-200 ps time range (presented in Fig. S2 (a)) were chosen to obtain a single-exponential fit, likewise, was done in the manuscript (see Fig. 4 (a)). As can be seen, the slope of

the power dependence for the 6 MLs sample (approx. 35 ps per nJ/cm^2) is flatter in comparison to the 50 MLs (approx. 50 ps per nJ/cm^2), indicating a weaker power dependence of the fast component of the thinnest barrier sample.

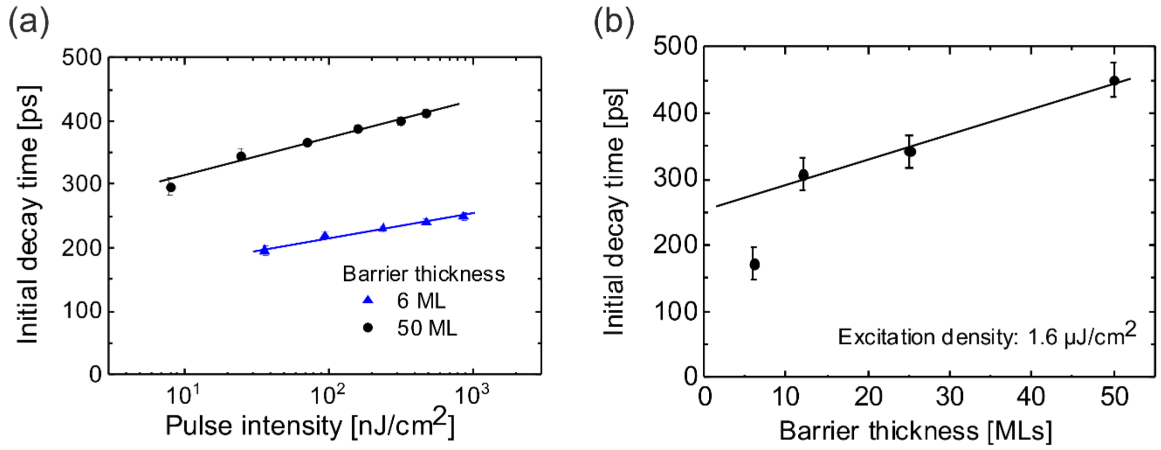

Fig. S2(a) Initial decay (0-200 ps) of the power dependent TR PL for the samples with 50 MLs (circles) and 6 MLs (blue triangles) barriers. (b) Initial decay times of all strictures with different barriers under. Error bars show a range of the decay that can be obtained under higher and lower excitation density.

One can also see the increase of the population for the sample with the thicker barrier that results in the longer decay. To understand the influence of this population effect on recombination we estimate the non-equilibrium carrier concentrations for 4.79 eV excitation. Absorption coefficients for both materials were assumed to be 200 000 cm^{-1} for 4.79 eV excitation, the reflectance of the GaN was taken as 18%[7]. Two situations were considered: (i) all carriers generated in the entire stack were transferred to the QWs and (ii) carrier pairs generated in the barriers do not contribute to the population in the QWs due to i.e. non-radiative recombination in the barriers. Assuming a pulse intensity of 240 nJ/cm^2, which corresponds to an excitation density of 20 W/cm^2, one obtains for the case (i) a carrier population given in Table S1.

Barrier thickness, MLs	Total thickness (QW; Barrier), nm	Total absorption	Total absorbed carriers in the stack, per cm^2	Mean carrier population per QW, per cm^2
50	2.6; 125	0.93	2.4×10^{11}	2.4×10^{10}
25	2.6; 60	0.72	1.8×10^{11}	1.8×10^{10}
12	2.6; 30	0.49	1.3×10^{11}	1.3×10^{10}
6	2.6; 15	0.30	7.7×10^{10}	7.7×10^{9}

Table S1 Non-equilibrium carrier populations per excitation pulse in the different structures. For the estimation, it was assumed that all carriers generated by both - barriers and QWs contribute to the population in the QWs.

Carrier population densities exclusively ascribed to the absorption in the QWs (case ii) are listed in Table S2. Thicker barriers reduce the pulse intensity to which the following QWs are exposed, thus, leading to a smaller mean carrier population.

Barrier thickness, MLs	Total thickness (QW; Barrier), nm	Absorption only in the QW	Total absorbed carriers in the QWs, per cm^2	Mean carrier population per QW, per cm^2
50	2.6; 125	0.0165	4.2×10^{9}	4.2×10^{8}
25	2.6; 60	0.0279	7.2×10^{9}	7.2×10^{8}
12	2.6; 30	0.0371	9.5×10^{9}	9.5×10^{8}
6	2.6; 15	0.0432	1.1×10^{10}	1.1×10^{9}

Table S2 Non-equilibrium carrier populations per excitation pulse in the different structures. For the estimation, it was assumed that carriers exclusively generated by the QWs contribute to their population. Carriers created in the barriers are considered as lost by non-radiative recombination.

Comparing the mean carrier population per QW given in the last columns of both tables, one can see that the carrier density of the 6 MLs sample either decreases by a factor of 3 (as shown in Table S1) or increases by a factor of 3 (see Table S2) with respect to the 50 MLs sample. Therefore, considering the decay time of the 6 MLs sample, at one specific excitation density, we still see a significant difference to the decay times of the 50 MLs SL even if we assume an excitation density increased or decreased by a factor of 3. Thus, we have chosen two excitation powers for the TRPL experiment (see Fig. S4 (a) in the manuscript) energies differing by one order of magnitude to account for the cases (i) and (ii) described here.

In Fig. S2 (b), decay times are shown under an excitation power of 10 mW. The enlarged “error” bars (±25 ps) of the decay times, taken from an average from the measurements, represent the difference to the initial decay times when the excitation power is reduced or increased by a factor of 3 (see slopes in Fig. S2 (a)). It is obvious, that the initial decay times of samples with thick barriers (above 12 MLs) follow a similar linear trend towards thickness of the barrier. While the 6 MLs SL clearly shows a faster decay time, pointing to an inherent difference in the recombination behavior for this sample, which cannot be caused by the different carrier densities in the QWs.

DFT calculations of the bandgap energies and effective masses

The difference of the bandgaps, with respect to GaN as a function of the GaN barrier thickness obtained from our DFT calculations is displayed in Fig. S3 (a). In the same figure, we show the measured SL peak emission (see Table 1 in the manuscript) shifts with respect to the GaN PL emission energy (3.46 eV). The calculated SL bandgaps and the measured PL emission peaks exhibit a similar trend. In both cases, the slope of the redshift becomes steeper for barriers thinner than approximately 8 MLs. Discrepancies on the absolute scale (~250 meV) between the experimental PL and the calculated bandgap shift are tolerable in view of the fact that in DFT we consider ML laterally periodic alloy with $In_{0.25}Ga_{0.75}N$ QWs.

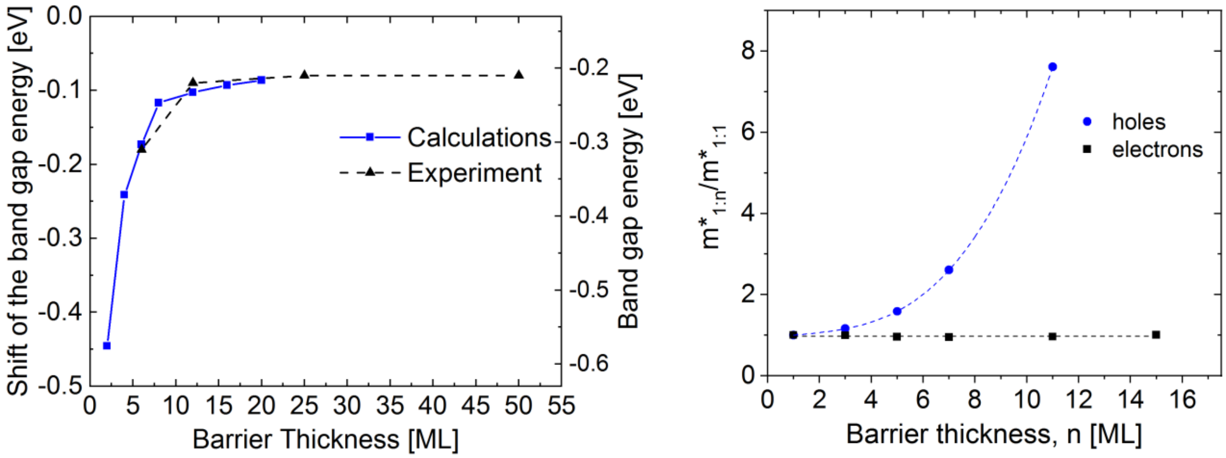

Fig. S3 (a) Calculated (blue squares) bandgap and experimental peak emission energy (triangles) from all samples of the series. The calculated bandgap of GaN is 3.09 eV. (b) Effective masses of electrons and holes along <0001> for different SL thickness normalized to the effective masses of the 1:1 SL. The dashed curves are guides to the eye. Within the accuracy of our calculations the states at the VBM are dispersionless for barriers thicker than 11 MLs (see text).

Next, we have estimated the effective masses of both carriers by means of DFT. The different distribution of the charge carrier densities of the electrons and holes strongly influence the corresponding effective masses and, hence, carriers' mobility. From the dispersion of the lowest unoccupied and highest occupied states around the Γ point, we have calculated the effective masses of electrons and holes along the <0001> direction. It should be mentioned here, that due to the short length of the Brillouin zone of the thick SLs and the accuracy of 0.1 meV in the energy of the eigenstates, we were able to accurately calculate the effective masses of the holes for barriers thinner than 11 MLs. Within the aforementioned accuracy the hole bands for SLs with barriers thicker than 15 MLs are dispersionless. The effective masses normalized to the effective mass of the 1:1 SL are summarized in Fig. S3 (b). As can be clearly seen the electron effective masses are insensitive to the barrier width. In contrast, the hole masses perpendicular to the SL plane monotonously increase with the barrier width.

1. Bai, J., Wang, T. & Sakai, S. Influence of the quantum-well thickness on the radiative recombination of InGaN/GaN quantum well structures. *J. Appl. Phys.* **88,** 4729–4733 (2000).

2. Das Sarma, S., Jalabert, R. & Yang, S. R. E. Band-gap renormalization in semiconductor quantum wells. *Phys. Rev. B* **41,** 8288–8294 (1990).

3. Nagai, T., Inagaki, T. J. & Kanemitsu, Y. Band-gap renormalization in highly excited GaN. *Appl. Phys. Lett.* **84,** 1284–1286 (2004).

4. Takeuchi, T. *et al.* Quantum-Confined Stark Effect due to Piezoelectric Fields in GaInN Strained Quantum Wells. *Jpn. J. App. Phys.* **36,** L382–L385 (1997).

5. Kuroda, T. & Tackeuchi, A. Influence of free carrier screening on the luminescence energy shift and carrier lifetime of InGaN quantum wells. *J. Appl. Phys.* **92,** 3071–3074 (2002).

6. Gotoh, H., Tawara, T., Kobayashi, Y., Kobayashi, N. & Saitoh, T. Large piezoelectric effects on photoluminescence properties in lonm-thick InGaN quantum wells. *Pacific Rim Conf. Lasers Electro-Optics, CLEO - Tech. Dig.* **1,** 341 (2003).

7. Bertrand, Y., Divrechy, A. & Raisin, C. Reflectivity and band structure of SnSe2. *J. Phys. C Solid State Phys.* **10,** 4155–4161 (1977).
